# Recent finding and new technologies in nephrolitiasis: a review of the recent literature

**DOI:** 10.1186/1471-2490-13-10

**Published:** 2013-02-16

**Authors:** Marco Rosa, Paolo Usai, Roberto Miano, Fernando J Kim, Enrico Finazzi Agrò, Pierluigi Bove, Salvatore Micali

**Affiliations:** 1 Department of Urology, University of Modena, Via del Pozzo, 71-41124, Modena, Italy; 2 Department of Urology, University of Cagliari, Via Aurelio Nicolodi, 1 09123, Cagliari, Italy; 3 Department of Urology, University “Tor Vergata”, Rome, Italy; 4 Department of Urology, Denver Health Care Center, 777 Bannock Street, Denver, CO, 80204-4597, USA

**Keywords:** Nephrolithiasis, New technologies, Diagnostic procedures, Risk factors, Ureterorenoscopy, Robotic-assisted surgery, Shock wave lithotripsy

## Abstract

This review summarizes recent literature on advances regarding renal and ureteral calculi, with particular focus in areas of recent advances in the overall field of urolithiasis. Clinical management in everyday practice requires a complete understanding of the issues regarding metabolic evaluation and subgrouping of stone-forming patients, diagnostic procedures, effective treatment regime in acute stone colic, medical expulsive therapy, and active stone removal. In this review we focus on new perspectives in managing nephrolitihiasis and discuss recentadvances, including medical expulsive therapy, new technologies, and refinements of classical therapy such as shock wave lithotripsy, give a fundamental modification of nephrolithiasis management. Overall, this field appears to be the most promising, capable of new developments in ureterorenoscopy and percutaneous approaches. Further improvements are expected from robotic-assisted procedures, such as flexible robotics in ureterorenoscopy.

## Review

### Introduction

Treating patients with urolithiasis is part of the everyday urological practice. An excellent clinical management involves a complete knowledge of issues regarding metabolic evaluation and subgrouping of stone-forming patients, diagnostic procedures, an effective treatment regime in acute stone colic, medical expulsive therapy, and active stone removal. In the 1980s, results of revolutionary technology such as shock wave lithotripsy (SWL) dramatically changed the therapeutic panorama of lithiasis, while open surgery was disappearing. Today the most invasive procedure for patients with significant stone burden is percutaneous nephrolithotomy (PNL). Furthermore, over the past decade, profound advances in endoscope design, durability, and accessories revolutionized the field of minimally invasive therapy.

Here, we review recent advances in the management of stone disease. We have specifically focused on the following sections:

a) epidemiology and risk factors;

b) metabolic evaluation and medical therapy;

c) diagnostic procedures;

d) SWL;

e) surgery, endoscopic procedures, and robot assisted procedures.

### Epidemiology and risk factors

International epidemiological data suggest that the incidence and prevalence of stone disease is increasing [1]. Recent data analysis show a higher prevalence in white population and stronger associations of prevalent kidney stone disease with increased triglycerides, older age, and gallstone disease in African Americans compared to whites, whereas male gender showed stronger association in whites; a dramatic increase of prevalence in female populations is also observed [2]. There was a significant increase in the incidence of kidney stones in children between 1996 and 2007 [3].

Recent papers focused on the most prominent metabolic issues of urolithiasis affecting an ever increasing number of people in developed countries: obesity, diabetes mellitus, hyperuricemia, and metabolic syndrome [4-9]. All these pathologic entities are strongly correlated with stone-former patients. After calcium-rich diets were found not to correlate with increased risk of stone formation, whereas calcium and Vitamin D supplementation played a pivotal role in stone-former patients [10]. The protecting role of adequate diet characterized by a high intake of fluids, fruits, and vegetables, a low consumption of salt and protein and a balanced intake of calcium, fats, and carbohydrates constitutes an efficacious approach to the prevention and treatment of this illness [5,11].

### Metabolic evaluation and medical therapy

Identification of metabolic risk factors and correct interpretation of collected data play an important role in managing stone patients and preventing recurrence wherever possible. The new edition of the European Association of Urology (EAU) Guidelines on Urolithiasis includes a useful system of subgrouping stone-forming patients into different categories, based on the type of stone and the severity of symptoms of the disease and also includes a simplified overview of the principles of analytical work-up [12]. Other authors underlined the fundamental role of metabolic work-up in high-risk stone formers [13] and children [14,15]. Still controversial is the role of urology specialists in fields where the nephrologist often plays a major role.

Since patient compliance largely influences medical treatment outcomes, adequate patient information regarding drinking and dietary recommendations plays a major role [12,13,16]. Dietary and drinking advice should always be considered before any pharmacological therapy. Correct dietary regimes should never be abandoned even when a pharmacological approach is started.

Various therapeutic tools were used in order to reduce the risk of recurrent calcium stones, that may result in stabilization of stone disease and prevention of the need for further surgical procedures for stone removal [16,17]. Turk *et al.* gives a brilliant effort to summarize all the suggested treatments and recommendations [12].

#### Alkaline citrate

Alkalinizations of tubular cells is the most important factor that results in an increased citrate excretion with only a small fraction of citrate preparations excreted with urine. Citrate calcium chelation reduces ion-activity products of both calcium oxalate and calcium phosphate and inhibits growth and aggregation/agglomeration of these crystals [12]. Thus citrate dilate lithogenesis promotes urinary alcalinization (reducing uric acid supersaturation) and increases cystine solubility. Citrate supplementation plays a fundamental role particularly in patients with hypocitraturia, which constitutes 20% of all stone formers [12,18,19]. Various citrate preparations (sodium potassium citrate, potassium citrate, potassium magnesium citrate, potassium bicarbonate, and sodium bicarbonate) were known to reduce the risk in stone-former patients. Findings based on randomized studies show that potassium citrate has a greater potential for preventing recurrence than does sodium potassium citrate [20-26]. When oral intake of citrate preparations is unpleasant for the patient, lemon or orange juice could be a valuable option, the latter being a better alkalinising and citraturic agent [21-23]. Citrate supplementation is also useful to considerably decrease stone formation risk that is correlated with prolonged bed rest [20].

#### Thiazides and thiazide-like agents

After the initial report by Yendt in 1970 we have more than 30 years of clinical experience with thiazides for calcium stone prevention [27,28]. The aim of thiazide treatment is to reduce calcium excretion in hypercalciuric patients (which constitutes around half of stone formers). This effect is thought to be mediated by an increased reabsorption of calcium in the proximal and distal part of the nephron [27-29]. Idiopathic hypercalciuria is a common disorder in children and can present with a range of clinical presentations such as hematuria, voiding dysfunction, flank pain, abdominal pain, nephrolithiasis, urinary tract infection and decreased bone mineral density. Dietary modifications are often sufficient in the management of hypercalciuria. If the symptoms persist or a rare monogenic disorder is present, consideration should be given to medical treatment with a thiazide diuretic and/or citrate therapy [30]. Hydroclorothiazide is usually given at a 25–50 mg dosage once or twice daily. A supplementation with potassium salt (i.e., potassium citrate 3.5–7 mmol twice daily) is needed to counterbalance the thiazide-induced potassium loss and hypocitraturic effect [12,31,32]. Thiazide treatment has considerable metabolic side effects: unmasking normocalcaemic hyperparathyroidism, development of diabetes and gout, and erectile dysfunction contribute to a limited patient compliance (50–70%) and high dropout rate [12,16,33].

#### Allopurinol

A xantine-oxidase inhibitor that prevents uric acid production from purine, allopurinol is a commonly used and usually well tolerated anti-gout drug [34]. In urolithiasis patients, treatment is given to counteract the formation of calcium oxalate stones. Allopurinol use in this pathologic condition was introduced following demonstration of a relationship between hyperuricosuria and calcium oxalate stone formation. Allopurinol has been used clinically in patients with or without hyperuricosuria. During the 1980s, Miano *et al.*[35] performed a placebo-controlled study where treatment with allopurinol was given to hyperuricosuric, calcium oxalate stone formers. Results were favorable to the allopurinol group, where 75% of patients were free of recurrent stones compared with 45% of the placebo group. Other randomized studies where patients were not selected for hyperuricosuria found no effect on stone formation, thus recent published EAU Guidelines [12] suggest that allopurinol “might be useful for treating patients with hyperuricosuric calcium stone formation” but it “cannot be recommended for patients with other biochemical abnormalities”. A new potential pharmacologic therapy for recurrent stone disease is described by Goldfarb *et al.* Febuxostat, a nonpurine inhibitor of xanthine oxidase (also known as xanthine dehydrogenase or xanthine oxidoreductase) may have advantages over allopurinol and is being tested in a similar protocol, with the eventual goal of determining whether urate-lowering therapy prevents recurrent calcium stones [36]. The major drawback of allopurinol treatment is the occurrence of severe side effects reported with high doses. Adverse effects include Steven-Johnson or Lyell syndrome, vasculitis, hepatitis, and renal failure. Allopurinol should be discontinued immediately in case of cutaneous rush [34].

#### Phytotherapy

Various herbal preparations have been used in urolithiasis therapy since ancient times [37]. Grases *et al.* evaluated the antilithiasic activity of herbal extract and antioxidant flavonoids (catechin and epicatechin) in rats with ethylene glycol induced lithiasis. Herbal preparations and flavonoids showed the ability to prevent papillary and intratubular calcification in the kidney [38]. Phytotherapy was probably clinically efficacious in hastening stone expulsion (<8 mm) without any observed adverse events [39]. Other herbal preparations show efficacy in stone expulsion after SWL (see further).

### Diagnostic procedures and interventional radiology

#### Computed tomography

Non-contrast computed tomography (NCCT) has been introduced during recent years and has become the well-recognized gold standard and most clinically useful tool for diagnosis of urolithiasis [40-42]. One great advantage is its ability to detect alternative diagnoses and to identify uric acid and xanthine stones that are radiolucent on plain film. This method shows superior [43] specificity and sensitivity compared with Intra Venous Pyelography urography. Combined IVU and CT study allowed correct diagnosis of the underlying cause of delayed excretion or upper urinary tract dilatation in 97% of cases, reducing time and radiation [44].

Still controversial is its role during follow-up for treated urolithiasis patients and those on observation protocol. Potretzke and Monga [45] suggested that follow-up should be done with plain film radiography if the stone is radio-opaque. Surveillance in children, uric acid stone-formers, and cystine stone-formers should be performed with ultrasonography. Pediatric patients treated for symptomatic urolithiasis could have completed their evaluation and treatment without undergoing NCCT in nearly 90% of the cases [46].

The usefulness of plain radiography is still under debate. Lamb *et al.*[47] determined the proportion of stone patients in which management is altered by the interpretation of plain abdominal radiographs (KUB). They observed that on the basis of KUB findings a significant change in patient management occurred. Data shows that KUB offers a significant advantage in treatment planning once the diagnosis has been established by NCCT because of information it provides regarding radio-opacity as well stone size and visibility. Johnston *et al.* agree if the stone is visible on CT scout film, then the decision to use KUB for follow-up can be made. This minimizes radiation exposure and other costs [48]. But stone features cannot be delivered by NCCT alone. NCCT size estimation of distal ureteral stones versus their actual size was investigated by Kishore and coworkers [49]. Findings show that CT is a poor predictor of the largest stone dimension for distal calculi. Thus caution should be used in patient counseling on the rate of spontaneous passage. Experimental studies using dual energy CT images (ie, scanners that can simultaneously acquire images at different energies) try to offer a routine clinical practice to estimate urinary stone composition based on the density of all constituent voxels [50]. In recent study, the introduction of dual-energy computed tomography systems has significant and unique applications for urologists. Imaging data from these scanners can be used to evaluate composition of urinary calculi [51].

#### Ultrasound

Ultrasonography is a well-recognized diagnostic tool and is usually the first imaging modality during diagnostic work-out. Ultrasonography is furthermore a safe and useful option in both the pediatric and pregnant populations, for whom it constitutes the imaging modality of choice. Further studies refined the diagnostic usefulness of ultrasonography in the localization of distal ureter calculi by imaging transrectally and transvaginally [52,53]. Mitterberger *et al.* placed ultrasound transducers with three-dimensional and volume scanning capabilities, transvaginally in female patients and transrectally in males. The authors were able to exhibit stones with 100% sensitivity, improving diagnosis in patients examined with transabdominal ultrasound and intravenous urogram (IVU), which together had 81% sensitivity.

*In vitro* study is enveloping a promising modality to facilitate spontaneous clearance of kidney stones and increased clearance of residual stone fragments after surgical management. Shan *et al.* present a novel method and device to reposition kidney stones using ultrasound radiation force delivered by focused ultrasound and guided by ultrasound imaging. Feasibility of repositioning stones was investigated by implanting artificial and human stones into a kidney-mimicking phantom that simulated a lower pole and collecting system. During experiment, stones were located by ultrasound imaging and repositioned by delivering short bursts of focused ultrasound. Stones were seen to move immediately after delivering focused ultrasound and successfully repositioned from the lower pole to the collecting system [54].

#### Radiation exposure

Patients undergoing diagnostic imaging may receive excessive doses of radiation during initial diagnostic and follow-up evaluations. Renal collecting systems can be illustrated more precisely with the advent of multi-detector row CT through thinner slices, high speed acquisitions, and enhanced longitudinal spatial resolution resulting in improved reformatted coronal images. On the other hand, a significant increase in exposure to ionizing radiation, especially in the radiosensitive organs, such as the gonads, is a concern with the increased utilization of urinary tract CT [55]. Few studies investigated the effective radiation dose associated with an acute stone episode and short-term follow-up. Ferrandino *et al.* in a single-institution study found that 205 patients received a dose greater than 20 mSv. John *et al.* found a median radiation dose per stone episode of 5.3 mSv, with higher doses in those with renal stones and those who required CT scans and other interventions. Ferrandino suggests that urologists must be cognizant of the radiation exposure to patients and seek alternative imaging strategies to minimize radiation dosages during acute and long-term stone management. [56,57]. In the US, around 60 million CT scans are performed every year [43], raising concern about the amount of radiation delivered. Thus different lower-dose radiation protocols were proposed [56-60]. Results show a high efficacy of lower-dose CT. Unfortunately, studies defined standard and low-dose protocols differently. A standard protocol uses about 180 mAs and low-dose protocol would be performed with about 30 mAs. Furthermore, a major role is played by the slice thickness and therefore the patient’s time exposure. But low-dose protocols use thicker slices than standard protocols, raising the risk of failure in detecting smaller stones. Memarsadeghi *et al.* determined that overlapping 3–5 mm slices could be a sufficient parameter for detection of significant urinary stones [61]. Ciaschini *et al.* found no significant differences with low dose (−25% and −50%) examinations for the detection of calculi greater than 3 mm [62]. Jellison et. al and Jin *et al.* compared ultra low dose and conventional computerized tomography protocols for detecting distal ureteral calculi [63] and renal calculi [64] in a cadaveric model. Jellison’s ultra low dose computerized tomography protocols detected distal ureteral calculi in a fashion similar to that of conventional computerized tomography protocols in a cadaveric model. These protocols may decrease the radiation dose up to 95%. Jin decreased the tube charge from 100 to 30 mAs, resulted in similar detection of renal stones respect conventional CT. Dose reduction is also important in pediatric settings. The use of the 80 mA setting for all children and 40 mA for children weighing 50 kg or less does not significantly affect the diagnosis of pediatric renal stones [65].

### SWL

SWL has changed dramatically the management of urolithiasis since the early 1980s. Widespread use of the technology, development of smaller devices, modified indication, and the lower cost of the procedure revolutionized the approach to stone patients. The large amount of sessions performed in the last 25 years allowed for the collection of important data on the indications, contraindications, and adverse effects of the procedure. Krambeck *et al.*[66] collected data regarding diabetes and hypertension associated with SWL performed with a Dornier HM3 lithotripter. Hypertension incidence was significantly correlated with bilateral procedures, while diabetes was correlated with shock wave number and frequency. The authors suggest that unobserved micro-trauma on the pancreas and kidney could explain the incidence of diabetes and hypertension. On the other side recent study by Chew *et al.*[67] compare the prevalence of hypertension and diabetes mellitus (DM) in patients treated with an unmodified HM-3 lithotripter (USWL) and a second-generation modified HM-3 lithotripter (MSWL) 20 years ago at their centre in Vancouver with that in the provincial population. No association between lithotripsy and the development of either DM or hypertension in a multivariate analysis. They postulate that the development of renal calculi in our subjects is more indicative of an overall metabolic syndrome where there is increasing evidence that patients with kidney stones get hypertension and diabetes and vice-versa. The development of these diseases is not related to shockwave lithotripsy, but rather to a systemic metabolic dysfunction.

Lee *et al.* in 2011 propose SWL treatment at a frequency of 60 shocks/min yielded better outcomes, such as a lower number of SWL sessions, and had an increased success rate compared with SWL at 120 shocks/min. On the other hand, pretreatment did not impact renal injury. Therefore, SWL treatment at a frequency of 60 shocks/min could improve treatment efficacy more than that for SWL at 120 shocks/min. [68]. Mazzucchi *et al.* found no significant differences in the stone-free and complication rates were observed by reducing the total number of impulses from 4000 to 3000 and the frequency from 90 to 60 impulses per minute [69]*.* Chacko *et al.* favors a frequency reduction, arguing that 90 sw/min treatment gave better results in terms of stone fragmentation compared to 120 sw/min [70]. Further reduction of frequency (30 sw/min) showed a protective effect on renal vessels in an animal model [71]. Furthermore, Tham *et al.* observed optimal fragmentation by using a short delay time (20 μs) between shock waves [72].

In animal models, stepwise power increases (18–20–22 kV) during treatment gave better results in terms of stone comminution compared with power decreases or leveling (96.5% vs. 89% vs. 87.6%, respectively). Moreover, Willis *et al.* proposed a “pre-treatment” of the kidney with low-energy shock waves (12 kV) in order to reduce renal injury [73,74].

Nomograms were introduced by Kanao *et al.* correlating stone size, location, and numbers to predict stone-free rates after the procedure using a Dornier Lithotripter D [75]. Recently, Nakajiima and Kanao validated the nomograms, finding a remarkable area under the curve (AUC) value of 0.725 [76]. This remarkable effort to predict outcome was until now limited to the Dornier machine. Vakalopoulos [77] avoided this gap by developing a mathematical model to predict extracorporeal shockwave lithotripsy outcomes where predictive equations can be created for different lithotripters. Wiesenthal JD *et al.* developed a comprehensive nomogram to predict renal and ureteral stone shock wave lithotripsy outcomes, dependent on patient and stone related factors. This factors included stone location, were age, body mass index, stone size, mean stone density (p < 0.01) and skin to stone distance [78].

Shen *et al.* Perform a systematic review to assess the necessity and complications of DJ stenting before extracorporeal shock wave lithotripsy in the management of upper urinary stones. The systematic review suggested significant advantages of stenting before extracorporeal shock wave lithotripsy compared to *in situ* extracorporeal shock wave lithotripsy in terms of Steinstrasse. However, stenting did not benefit stone-free rate and auxiliary treatment after extracorporeal shock wave lithotripsy, and it induced more lower urinary tract symptoms [79]. El Assmi found that the presence of hydronephrosis does not affect success rates for distal ureteral stones but increases the number of treatments needed to obtain stone clearance [80]*.*

Recent studies showed the usefulness of CT imaging in predicting fragility and consequently outcomes after SWL. Such imaging could also offer a considerable amount of information regarding intrarenal anatomy (i.e., lower pole calyx orientation), stone location, and stone composition. Studies by Alon et Garcia Marchinena *et al.* attempt to characterize stone composition with CT in order to have an indication in the management of calculi suggesting a first-line endoscopic therapy instead of SWL [81,82].

Furthermore, the risk of SWL failure is significantly related to increased radiodensity (signal attenuation) both *in vitro* and *in vivo*. Cystine, calcium oxalate monohydrate, and brushite stones are least likely to be fragmented by SWL [83-86].

#### Post-SWL therapy

An extensive meta-analysis of medical therapies could be found in the paper by Shuller *et al.* and Micali and coworkers [16,87]. The latter investigated the role of *Phyllanthus niruri* (a plant belonging to the Euphorbiaceae family used in Brazilian folk medicine by patients with urolithiasis) in SWL and found a positive correlation with lower calyx stone expulsion [88].

The same author [89] and Zheng *et al.*[90] explore the efficacy of expulsive therapy using nifedipine or tamsulosin, both associated with ketoprofene, after SWL of ureteral stones. They found that nifedipine and ketoprofene association play a significant role in increasing stone free rates for the proximal and middle ureter (85.7% vs. 51.7%) and that tamsulosin and ketoprofen increase stone free rates in distal ureter stones (82.1% vs. 57.1%). Falahatkar S *et al.*[91] study the role of tamsulosin as adjunctive therapy after extracorporeal shock wave lithotripsy (ESWL) in 150 patients with 4–20 mm in diameter renal and ureteral stones. Th patients was shared 71 in control group and 70 in case group, treated with Tamsulosin: thei found a statistically significant difference in time of stone passage from onset of treatment (between 20th and 30th day in control group and between 10th and 20th day (50%) in case group after ESWL). Sighinolfi *et al.*[92] found that treatment with Tamsulosin after ESWL increases fragments expulsion rate of renal calculi also.

As seen above, citrate supplementation could play an important role in expulsive therapy after SWL. In a study conducted on 96 hypocitraturic children who underwent SWL potassium citrate showed a significant role in decreasing recurrence (7.6% in citrate arm vs. 34.6% in placebo) agglomeration of residual fragments [25].

#### SWL vs. URS

Debate over the most favorable method still continues and probably will continue for decades. Kijvikai *et al.*[93] try to offer some balanced consideration on the better treatment for distal ureteral stones. SWL and URS (ureteroscopic lithotripsy) are both considered to produce excellent stone free rates (86–90%), but stones >10 mm have better outcomes with endoscopy (73% vs. 67%). Aboumarzouk OM *et al.*[94] agree that, compared with ESWL, ureteroscopic removal of ureteral stones achieves a greater stone-free state, but with a higher complication rate and longer hospital stay. Furthermore, URS plays a unique role during pregnancy or in patients with uncontrolled blood coagulation [93,95].

In conclusion, SWL revolutionized urolithiasis therapy and is often the treatment of choice for many ureteral and renal stones. Moreover, SWL is related to low complication rates. But a balanced choice should always include patient consideration [96].

### Digital endoscopes

At the beginning of this century, ureteroscopy received a new impulse thanks to novel technological refinements such as miniaturization of scope profile, improved maneuverability, and optimized accessory instrumentation. Currently available flexible ureteroscopes have an average tip diameter of about 6.9–7.5 Fr and a mid-shaft diameter of 7.5–9.0 Fr and can be inserted in an intramural ureter without active dilation in most cases [97].

**Figure 1:**
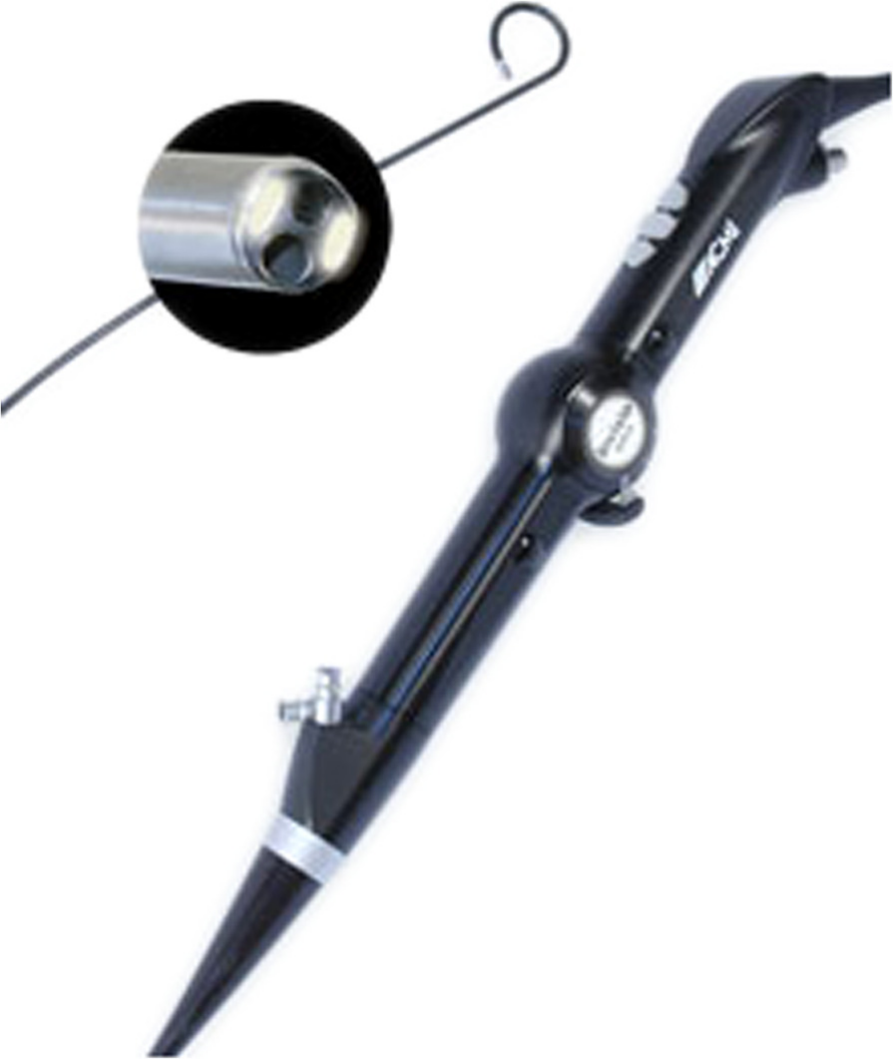
The DUR-D (Olympus) digital and flexible ureteroscope.

Image quality was also improved by incorporating an optical chip such as a CMOS (complimentary metal oxide semiconductor) or CCD (charge-couple device) at the tip of the ureteroscope together with distal LED light and image processing capabilities. Digital ureteroscopes eliminate the honeycomb effect, and deflection is comparable to traditional fiber-optic endoscopes. In September 2006, Gyrus-ACMI (Southboro, Massachusetts, USA) was the first to introduce a ureteroscope incorporating this technology: the DUR-D ureteroscope (Figure 1). Preliminary reports indicate that the new-generation flexible ureterorenoscopes are more durable than previous ones [98,99]. All these enthusiastic reports should be counterbalanced by an awareness of some disadvantages of the new-generation endoscopes. Rigid and flexible digital ureteroscopes are larger in diameter compared to their analog counterparts, and digital technology has higher costs. Thus more research is necessary to evaluate the true advantage of digital technology for ureteroscopy [100,101]. Undoubtedly, images produced by digital endoscopes such as DUR-D are of outstanding quality.

Deflection capability is also an important issue. The Storz FlexX2 Wolf Viper allows a 270° deflection in both directions, while the Olympus P5 allows 270° in one direction and 180° in the other. The DUR 8-elite ureteroscope (ACMI) was the first to offer dual primary and secondary active tip deflection that totals 270°. Preliminary reports suggest that secondary deflection is necessary in approximately 20–29% of cases [102-104], particularly with regard to lower pole access. Although expensive, the holmium: YAG laser is actually the best intracorporeal lithotripter for the ureter and the benchmark for other energy sources [105-107].

#### Accessories device

**Figure 2:**
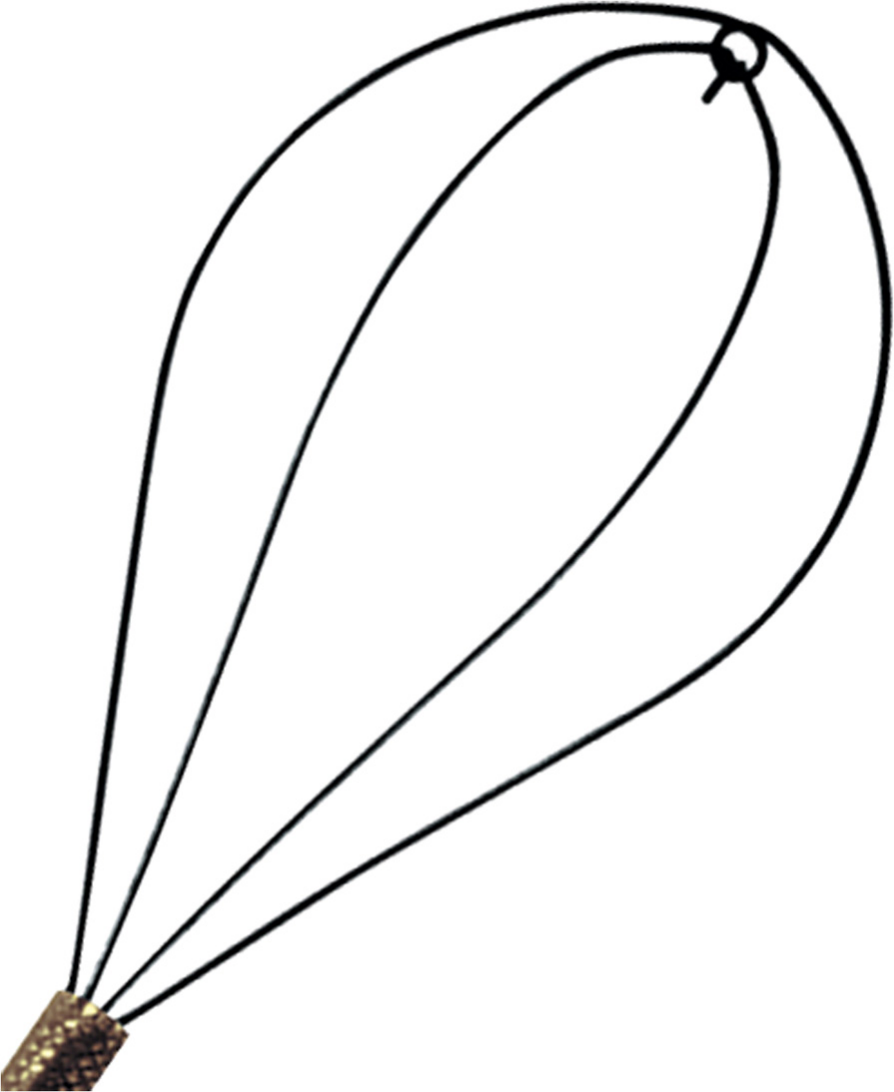
Cook N-Circle nitinol basket.

**Figure 3:**
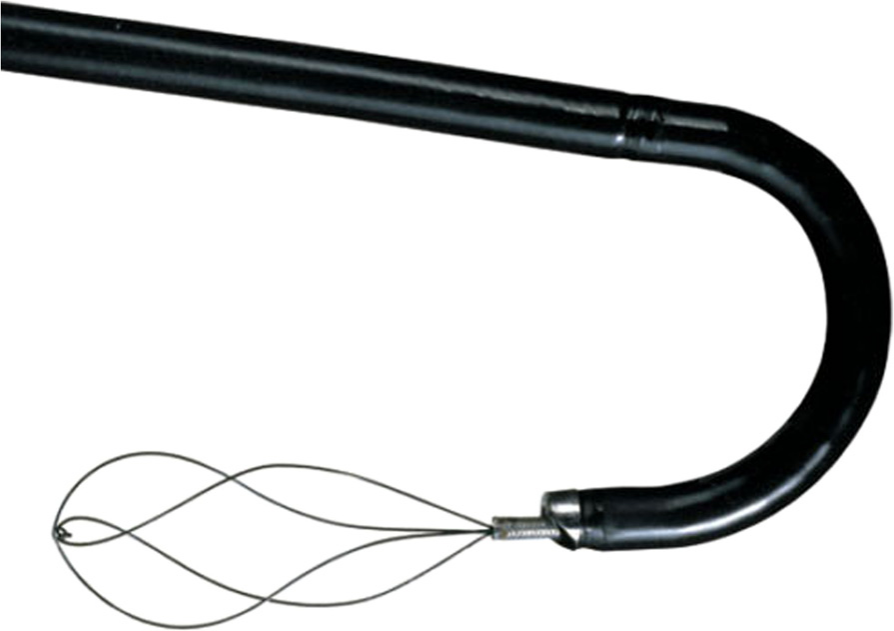
Cook N-Circle nitinol basket on flexible ureteroscope.

The ideal basket should be flexible, durable, atraumatic, easily deployed/disengaged/disassembled, and of minimal impact on fluid inflow and tip deflection [108]. Thus, the ideal basket simply does not exist until now. Despite marketing efforts to introduce the “perfect” basket, comparison of four popular basket designs suggested that the more complex wire configurations and deflection capabilities offered no advantage over the simple Cook N-Circle nitinol basket [108,109] (Figures 2 and 3).

### PNL

International epidemiological data suggest that the incidence and prevalence of stone disease is increasing and the number of diagnoses and procedures relating to kidney stone disease has increased significantly in the last 10 years in the UK [1]. Since the introduction of SWL during the 1980s and development of endoscopic techniques, open surgery is rapidly disappearing. Nowadays PNL represents the most invasive procedure for urolithiasis in significant stone burden patients. In the US and UK, PNL experienced a rapid increase while open surgery is showing a dramatic decreas [1,110].

Percutaneous access is the method of choice in staghorn and complex renal stones with diameters >2 cm and for lower pole calculi with diameters >1 cm [111]. Debates over patient position (prone or supine) and SWL efficacy versus PNL take place in many journals. PNL is indicated as the first-choice treatment in staghorn and complex renal stones >2 cm and for lower calyx stones >1 cm [112].

#### Patient position

Recent studies proposed and popularized the Valdivia Uria supine position for PNL. Valdivia Uria’s original paper dated to 1987 and more than 557 have been performed since then. According to the author, the advantages of the position are a direct and easily access to anterior oriented calyces, easier access to the bladder, and better stone free rates in comparison to the prone position [113]. A recent review summarized the arguments for and against prone and supine percutaneous nephrolithotomy: the prone position is associated with a decrease in the cardiac index and an increase in pulmonary functional residual capacity. An increased risk of liver and spleen injury exists for upper pole puncture with the patient supine. Potential injury to the colon is greatest during prone lower pole access. A greater surface area for percutaneous access exists with the patient prone. The supine position decreases surgeon radiation exposure and promotes spontaneous stone drainage during the procedure. Two comparative series show that the supine position is associated with significantly shorter operative time. In contrast, noncomparative case series suggest decreased operative time and blood loss when treating staghorn calculi with the patient prone [114].

The supine position is also indicated in a high American Society of Anesthesiologists (ASA) score [115]. Cracco *et al.* and Kawahara *et al.* propose ECIRS (Endoscopic Combined IntraRenal Surgery) is a new way of affording PNL in a modified supine position, approaching antero-retrogradely to the renal cavities, and exploiting the full array of endourologic equipment. Supine PNL and ECIRS are not superior to prone PNL in terms of urological results, but guarantee undeniable anesthesiological and management advantages for both patient and operators. In particular, ECIRS requires from the surgeon a permanent mental attitude to synergy, standardized surgical steps, versatility and adherence to the ongoing clinical requirements. ECIRS can be performed also in particular cases, irrespective to age or body habitus. The use of flexible endoscopes during ECIRS contributes to minimizing radiation exposure, hemorrhagic risk and post-PNL renal damage [116,117].

#### Percutaneous access

The debate over access numbers still continue. The authors suggest that higher stone free rates could be easily obtained with the use of flexible nephroscopes during the PNL [118,119]. Wong *et al.* reported a stone free rate of 95% in patients treated with a single percutaneous tract with flexible nephroscopy. The use of a single tract with high stone free rates seems the best achievement in terms of minimal invasiveness and the benchmark for procedure comparison [120]. Akman *et al.* consider the impact of PCNL using either single or multiple access tracts on renal function, finding similar results. By the way PCNL with multiple accesses is a highly successful alternative with considerable complication rates in the management of staghorn calculi [121]. Li NL *et al.* propose percutaneous nephrolithotomy through the upper pole calix. The access allows greater stone clearance rate due to its easy access into the intrarenal collecting system and can be an ideal approach for PCNL for complicated renal calculi [122].

#### PNL under local anesthetics

PNL under local anesthesia is a very attractive method in order to minimize procedure morbidity. Indication should be very strict and must exclude staghorn calculi patients, previous renal surgery, and stone burden >3.5 cm. Local anesthesia should also include percutaneous tract and renal parenchyma to achieve post-operative pain control [123,124].

#### Robotic surgery

Robot-assisted laparoscopic surgery with the daVinci system is immensely popular among urologists. However some of the earliest experiences with robotics in urology were developed by italian group of Bove in the last ’90. They performed different kind of procedures (spermatic vein ligation, retroperitoneal renal biopsy, simple nephrectomy and pyeloplasty) with the help of two robots: AESOP for the orientation of the laparoscope and PAKY to perform the percutaneous access [125]. Robot-assisted surgery is now well-established in Urology and although not currently regarded as a ‘gold standard’ approach for any urological procedure, it is being increasingly used for index operations of the prostate, kidney and bladder [126]. Fine movement control and drift-free maintenance of the endoscope distal tip is typically insufficient using manual control to perform complex procedures. The aim of robotic surgery is to allow safer and more homogeneous outcomes with less variability in surgeon performance and reduced occupational radiation exposure. Desai *et al.* tested on animals a new flexible robotic system for performing retrograde intrarenal surgery [127] and his initial clinical experience on 18 patient was encouraging: all procedures were technically successful without conversion to manual ureteroscopy and the complete stone clearance rate at 2 and 3 months was 56% and 89%, respectively. At 3 months all patients had stable renal function and unobstructed drainage [128]. According to the authors, potential advantages of the technique include increased range of motion, instrument stability, and improved ergonomics.

The nascent field of flexible robotics appears to be promising. Refinements in software and hardware are needed to allow these systems to be used for natural orifice transluminal surgery. A significant advance in robotic surgery came from URobotics Laboratory at Johns Hopkins (Baltimore, MD, USA), which recently developed the AcuBot robot. This device is a fully actuated driver for needle insertion, spinning, release, and force measurement. This provides an additional needle support guide in close proximity to the skin entry point. The device is the first promising step to a future clinical application of robotic guided percutaneous renal access [129].

#### “News from the past”

Recent research by Mariani assesses the feasibility of ureteroscopic monotherapy of renal calculi >2 cm. Lithotripsy was performed in 75 patients with a single deflection flexible ureteroscope and predominantly electrohydraulic lithotripsy; laser drilling was employed to weaken very hard stones. Stone free status was achieved in 96% of patients [130]. Recently, similar results was obtained by Hyams *et al.*: one hundred and twenty patients underwent URS/holmium laser lithotripsy for renal stones of 2 to 3 cm. One hundred and one (84%) patients underwent single-stage procedures.

## Conclusion

Urolithiasis is a growing problem in industrialized countries and is often correlated to typical Western pathologies and habits such as diabetes, hypertension, high purine intakes, obesity, and metabolic syndrome. Beside drug treatment, in recent years medical therapies incorporating herbal components known for centuries have been investigated. Data show that it offers new advantage in stone clearance after SWL or in spontaneous stone expulsion. NCCT is nowadays the most useful clinical tool in stone patients. Among all the minimally invasive stone treatments, SWL is always the less invasive one, and stone free rates with SWL are lower than with more invasive treatments. Therapy choice should include these considerations. Development of new technologies offers further advances in well-standardized procedures such as PNL and SWL. Robotics seems the most promising field capable of new developments in ureterorenoscopy and percutaneous approaches.

## Competing interests

The authors declare that they have no competing interests.

## Authors’ contributions

All authors read and approved the final manuscript.

## Pre-publication history

The pre-publication history for this paper can be accessed here:

http://www.biomedcentral.com/1471-2490/13/10/prepub

## References

[B1] Turney BW Trends in urological stone disease BJU Int 2011 24 6 382 386 10.1111/j.1464-410X.2011.10495.x 21883851

[B2] Akoudad S Correlates of kidney stone disease differ by race in a multi-ethnic middle-aged population: the ARIC study Prev Med 2010 51 5 416 420 10.1016/j.ypmed.2010.08.011 20801154 PMC2964449

[B3] Sas DJ Increasing incidence of kidney stones in children evaluated in the emergency department J Pediatr 2010 157 1 132 137 10.1016/j.jpeds.2010.02.004 20362300

[B4] Chen HS Increased risk of urinary tract calculi among patients with diabetes mellitus-a population-based cohort study Urology 2011 42 8 432 435 10.1016/j.urology.2011.07.1431 22119251

[B5] Frassetto L Treatment and prevention of kidney stones: an update Am Fam Physician 2011 84 11 1234 1242 22150656

[B6] Del Valle EE Metabolic diagnosis in stone formers in relation to body mass index Urol Res 2011 38 7 123 127 10.1007/s00240-011-0392-8 21660441

[B7] Maalouf NM Metabolic syndrome and the genesis of uric acid stones J Ren Nutr 2011 21 1 128 131 10.1053/j.jrn.2010.10.015 21195936 PMC3053068

[B8] Hurtes X Hyperuricemia and uro-nephrological disorders Presse Med 2011 40 9 Pt 1 865 868 21684106 10.1016/j.lpm.2011.05.006

[B9] Jeong IG Association between metabolic syndrome and the presence of kidney stones in a screened population Am J Kidney Dis 2011 58 3 383 388 10.1053/j.ajkd.2011.03.021 21620546

[B10] Wallace RB Urinary tract stone occurrence in the Women’s health initiative (WHI) randomized clinical trial of calcium and vitamin D supplements Am J Clin Nutr 2011 94 1 270 277 10.3945/ajcn.110.003350 21525191 PMC3127502

[B11] Meschi T Lifestyle recommendations to reduce the risk of kidney stones Urol Clin North Am 2011 38 3 313 320 10.1016/j.ucl.2011.04.002 21798393

[B12] Turk C EAU guidelines on urolithiasis 2011 editon 2011

[B13] Park S Medical management of urinary stone disease Expert Opin Pharmacother 2007 8 8 1117 1125 10.1517/14656566.8.8.1117 17516875

[B14] Dogan HS Management of pediatric stone disease Curr Urol Rep 2007 8 2 163 173 10.1007/s11934-007-0067-8 17303023

[B15] Sarica K Effect of potassium citrate therapy on stone recurrence and regrowth after extracorporeal shockwave lithotripsy in children J Endourol 2006 20 11 875 879 10.1089/end.2006.20.875 17144854

[B16] Micali S Medical therapy of urolithiasis J Endourol 2006 20 11 841 847 Rewiew 10.1089/end.2006.20.841 17144848

[B17] Zilberman DE Long-term results of percutaneous nephrolithotomy: does prophylactic medical stone management make a difference? J Endourol 2009 23 10 1773 1776 10.1089/end.2009.0118 19530951

[B18] Kurtz MP Dietary therapy for patients with hypocitraturic nephrolithiasis Nat Rev Urol 2011 8 3 146 152 10.1038/nrurol.2011.9 21321574

[B19] Rodgers A Evening primrose oil supplementation increases citraturia and decreases other urinary risk factors for calcium oxalate urolithiasis J Urol 2009 182 6 2957 2963 10.1016/j.juro.2009.08.021 19846138

[B20] Zerwekh JE Odvina CV Wuermser LA Pak CY Reduction of renal stone risk by potassium-magnesium citrate during 5 weeks of bed rest J Urol 2007 177 6 2179 2184 10.1016/j.juro.2007.01.156 17509313

[B21] Odvina CV Comparative value of orange juice versus lemonade in reducing stone forming risk Clin J Am Soc Nephrol 2006 1 6 1269 1274 10.2215/CJN.00800306 17699358

[B22] Kang DE Sur RL Haleblian GE Fitzsimons NJ Borawski KM Preminger GM Long-term lemonade based dietary manipulation in patients with hypocitraturic nephrolithiasis J Urol 2007 177 4 1358 1362 10.1016/j.juro.2006.11.058 17382731

[B23] Zuckerman JM Hypocitraturia: pathophysiology and medical management Rev Urol 2009 11 3 134 144 19918339 PMC2777061

[B24] Caudarella R Urinary citrate and renal stone disease: the preventive role of alkali citrate treatment Arch Ital Urol Androl 2009 81 3 182 187 19911682

[B25] Lojanapiwat Alkaline citrate reduces stone recurrence and regrowth after shockwave lithotripsy and percutaneous nephrolithotomy Int Braz J Urol 2011 37 5 611 616 10.1590/S1677-55382011000500007 22099273

[B26] Singh SKBJU Medical therapy for calculus disease Int 2011 107 3 356 368 10.1111/j.1464-410X.2010.09802.x 21244607

[B27] Yendt ER Renal calculi CMAJ 1970 102 5 479 489 5438766 PMC1946599

[B28] Yendt ER Commentary: renal calculi twenty years later J Lithotripsy Stone Dis 1990 2 164 172

[B29] Grieff M Diuretics and disorders of calcium homeostasis Semin Nephrol 2011 31 6 535 541 10.1016/j.semnephrol.2011.09.008 22099510

[B30] Srivastava T Diagnosis and management of hypercalciuria in children Curr Opin Pediatr 2009 21 2 214 219 10.1097/MOP.0b013e3283223db7 19307900

[B31] Vigen R Thiazides diuretics in the treatment of nephrolithiasis: are we using them in an evidence-based fashion? Int Urol Nephrol 2011 43 3 813 819 Epub 2010 Aug 25 10.1007/s11255-010-9824-6 20737209 PMC3229098

[B32] Reilly RF The evidence-based use of thiazide diuretics in hypertension and nephrolithiasis Clin J Am Soc Nephrol 2010 5 10 1893 1903 Epub 2010 Aug 26 10.2215/CJN.04670510 20798254

[B33] Huen SC Goldfarb DS Adverse metabolic side effects of thiazides: implications for patients with calcium nephrolithiasis J Urol 2007 177 4 1238 1243 10.1016/j.juro.2006.11.040 17382697

[B34] British National Formulary 2004 UK: BMJ Publication Group London

[B35] Miano L Petta S Galatioto GP Gallucci M A placebo controlled double-blind study of allopurinol in severe recurrent idiopathic renal lithiasis Urolithiasis and related clinical research New York 1985 New York: Plenum Press 521 524

[B36] Goldfarb DS Potential pharmacologic treatments for cystinuria and for calcium stones associated with hyperuricosuria Clin J Am Soc Nephrol 2011 6 8 2093 2097 Epub 2011 Jul 14 10.2215/CJN.00320111 21757641 PMC3156434

[B37] Tkachuk VN Phytotherapy in the treatment of ureteral calculi Urologiia 2009 12 3 13 15 19670808

[B38] Grases F Prieto RM Phytotherapy and renal stones: the role of antioxidants. A pilot study in wistar rats Urol Res 2008 in press 10.1007/s00240-008-0165-1 19066877

[B39] Singh I Prospective randomized clinical trial comparing phytotherapy with potassium citrate in management of minimal burden (≤8 mm) nephrolithiasis Urol Ann 2011 3 2 75 81 10.4103/0974-7796.82172 21747596 PMC3130482

[B40] Dhar M Imaging in diagnosis, treatment, and follow-up of stone patients Adv Chronic Kidney Dis 2009 16 1 39 47 10.1053/j.ackd.2008.10.005 19095204

[B41] Carter MR Renal calculi: emergency department diagnosis and treatment Emerg Med Pract 2011 13 7 1 17 22164398

[B42] Mandeville JA Imaging evaluation in the patient with renal stone disease Semin Nephrol 2011 31 3 254 258 10.1016/j.semnephrol.2011.05.006 21784274

[B43] Shine S Urinary calculus: IVU vs CT renal stone? A critically appraised approach Abdom Imaging 2008 33 9 41 43 17786506 10.1007/s00261-007-9307-0

[B44] Sebastià C Usefulness of computed tomography performed immediately after excretory urography in patients with delayed opacification or dilated upper urinary tract of unknown cause Abdom Imaging 2011 48 5 81 87 10.1007/s00261-011-9771-4 21748467

[B45] Potretzke AM Monga M Imaging modalities for urolithiasis: impact on management Curr Opin Urol 2008 18 199 204 10.1097/MOU.0b013e3282f46b11 18303544

[B46] Wallis MC Are stone protocol computed tomography scans mandatory for children with suspected urinary calculi? Int Braz J Urol 2011 37 5 681 682 Division of Pediatric Urology, University of Utah, Salt Lake City, Utah, USA 10.1590/S1677-55382011000500033 21722946

[B47] Lamb AD Wines MD Mousa S Tolley DA Plain radiography still is required in the planning of treatment for urolithiasis J Endourol 2008 22 10 2201 2205 10.1089/end.2008.9716 18937584

[B48] Johnston R Comparison of kidney-ureter-bladder abdominal radiography and computed tomography scout films for identifying renal calculi BJU Int 2009 104 5 670 673 10.1111/j.1464-410X.2009.08542.x 19694714

[B49] Kishore TA Estimation of size of distal ureteral stone: non-contrast CT scan versus actual size Urology 2008 72 4 761 764 10.1016/j.urology.2008.05.047 18701151

[B50] Boll DT Patil NA Paulson EK Merkle EM Simmons WN Pierre SA Preminger GM Renal stone assessment with dual-energy multidetector CT and advanced postprocessing techniques: improved characterization of renal stone composition–pilot study Radiology 2009 250 3 813 820 10.1148/radiol.2503080545 19244048

[B51] Park J Dual-energy computed tomography applications in uroradiology Curr Urol Rep 2011 16 3 178 183 10.1007/s11934-011-0226-9 22068585

[B52] Yang J Yang S Hsu H Huang W Transvaginal sonography in the morphological and functional assessment of segmental dilation of the distal ureter Ultrasound Obstet Gynecol 2006 27 449 451 10.1002/uog.2719 16514622

[B53] Mitterberger M Pinggera G Maier E Value of 3-dimensional transrectal/transvaginal sonography in diagnosis of distal ureteral calculi J Ultrasound Med 2007 26 19 27 17182705 10.7863/jum.2007.26.1.19

[B54] Shah A Novel ultrasound method to reposition kidney stones Urol Res 2010 38 6 491 495 Epub 2010 Oct 22 10.1007/s00240-010-0319-9 20967437 PMC3087440

[B55] Sung MK Current status of low dose multi-detector CT in the urinary tract World J Radiol 2011 3 11 256 265 10.4329/wjr.v3.i11.256 22132296 PMC3226959

[B56] John BS Patel U Anson K What radiation exposure can a patient expect during a single stone episode? J Endourol 2008 22 3 419 422 10.1089/end.2007.0268 18355136

[B57] Ferrandino MN Bagrodia A Pierre SA Scales CD Jr Rampersaud E Pearle MS Dual energy computed tomography with advanced postimage acquisition data processing: improved determination of urinary stone composition J Endourol 2010 24 3 347 54 10.1089/end.2009.0193 20105031

[B58] Preminger GM Radiation exposure in the acute and short-term management of urolithiasis at two academic centers J Urol 2009 181 2 668 672 10.1016/j.juro.2008.10.012 19100573

[B59] Poletti P Platon A Rutschmann O Low-dose versus standard-dose CT protocol in patients with clinically suspected renal colic AJR Am J Roentgenol 2007 188 927 933 10.2214/AJR.06.0793 17377025

[B60] McCollough C Bruesewitz M Kofler J CT dose reduction and dose managementtools: overview of available options Radiographics 2006 26 503 512 10.1148/rg.262055138 16549613

[B61] Memarsadeghi M Heinz-Peer G Helbich TH Unenhanced multidetector row CT in patients suspected of having urinary stone disease: effect of section width on diagnosis Radiology 2005 235 530 536 10.1148/radiol.2352040448 15758192

[B62] Ciaschini MW Remer EM Baker ME Lieber M Herts BR Urinary calculi: radiation dose reduction of 50% and 75% at CT–effect on sensitivity Radiology 2009 251 1 105 111 10.1148/radiol.2511081084 19251939

[B63] Jellison FC Smith JC Heldt JP Spengler NM Nicolay LI Ruckle HC Koning JL Millard WW Jin DH Baldwin DD Effect of low dose radiation computerized tomography protocols on distal ureteral calculus detection J Urol 2009 182 2762 2767 10.1016/j.juro.2009.08.042 19837431

[B64] Jin DH Effect of reduced radiation CT protocols on the detection of renal calculi Radiology 2010 255 1 100 107 10.1148/radiol.09090583 20308448

[B65] Karmazyn B Frush DP Applegate KE Maxfield C Cohen MD Jones RP CT with a computer-simulated dose reduction technique for detection of pediatric nephroureterolithiasis: comparison of standard and reduced radiation doses Am J Roentgenol 2009 192 1 143 149 10.2214/AJR.08.1391 19098193

[B66] Krambeck AE Diabetes mellitus and hypertension associated with shock wave lithotripsy of renal and proximal ureteral stones at 19 years of follow up J Urol 2006 175 5 1742 1747 10.1016/S0022-5347(05)00989-4 16600747

[B67] Chew BH Twenty-year prevalence of diabetes mellitus and hypertension in patients receiving shock-wave lithotripsy for urolithiasis BJU Int 2011 22 4 268 274 10.1111/j.1464-410X.2011.10291.x 21635683

[B68] Lee JY Evaluation of the optimal frequency of and pretreatment with shock waves in patients with renal stones Korean J Urol 2011 52 11 776 781 10.4111/kju.2011.52.11.776 22195268 PMC3242992

[B69] Mazzucchi E Comparison between two shock wave regimens using frequencies of 60 and 90 impulses per minute for urinary stones Clinics (Sao Paulo) 2010 65 10 961 965 10.1590/S1807-59322010001000006 21120294 PMC2972613

[B70] Chacko J Does a slower treatment rate impact the efficacy of extracorporeal shock wave lithotripsy for solitary kidney or ureteral stones? J Urol 2006 175 4 1370 1373 10.1016/S0022-5347(05)00683-X 16515999

[B71] Evan AP Renal injury during shock wave lithotripsy is significantly reduced by slowing the rate of shock wave delivery BJU Int 2007 100 3 624 628 10.1111/j.1464-410X.2007.07007.x 17550415

[B72] Tham LM Enhanced kidney stone fragmentation by short delay tandem conventional and modified lithotriptor shock waves: a numerical analysis J Urol 2007 178 1 314 319 10.1016/j.juro.2007.03.009 17499770

[B73] Maloney ME Progressive increase of lithotripter output produces better in vivo stone comminution J Endourol 2006 20 9 603 606 10.1089/end.2006.20.603 16999607 PMC1931482

[B74] Willis LR Prevention of lithotripsy- induced renal injury by pretreating kidneys with low –energy shock waves J Am Soc Nephrol 2006 17 3 663 673 10.1681/ASN.2005060634 16452495

[B75] Kanao K Preoperative nomograms for predicting stone-free rate after extracorporeal shock wave lithotripsyj Urol 2006 176 1453 1457 10.1016/j.juro.2006.06.089 16952658

[B76] Nakajima Y Kanao K Current topics in the management of urinary tract stones; preoperative nomograms for predicting stone-free rate after ESWL Proceedings of 29th annual jackson hole urologic conference

[B77] Vakalopoulos I Development of a mathematical model to predict extracorporeal shockwave lithotripsy outcome J Endourol 2009 23 6 891 897 10.1089/end.2008.0465 19441881

[B78] Wiesenthal JD A clinical nomogram to predict the successful shock wave lithotripsy of renal and ureteral calculi J Urol 2011 186 2 556 562 10.1016/j.juro.2011.03.109 21684557

[B79] Shen P Use of ureteral stent in extracorporeal shock wave lithotripsy for upper urinary calculi: a systematic review and meta-analysis J Urol 2011 186 4 1328 1335 10.1016/j.juro.2011.05.073 21855945

[B80] El-Assmy A Impact of the degree of hydronephrosis on the efficacy of in situ extracorporeal shock-wave lithotripsy for proximal ureteral calculi Scand J Urol Nephrol 2007 41 208 213 10.1080/00365590601068892 17469029

[B81] Alon Z New concepts in shock wave lithotripsy Urol Clin N Am 2007 34 375 382 10.1016/j.ucl.2007.07.002 17678987

[B82] García Marchiñena P CT SCAN as a predictor of composition and fragility of urinary lithiasis treated with extracorporeal shock wave lithotripsy in vitro Arch Esp Urol 2009 62 3 215 222 discussion 222 19542594 10.4321/s0004-06142009000300007

[B83] Leycamm L Observations on intrarenal geometry of the lower caliceal system in relation to clearance of stone fragments after extracorporeal shockwave lithotripsy J endourol 2007 21 4 386 392 10.1089/end.2006.0292 17451327

[B84] Madaan S Limitations of extracorporeal shock wave lithotripsy Curr Opin urol 2007 17 2 109 113 10.1097/MOU.0b013e32802b70bc 17285020

[B85] Sapozhnikov OA A mechanistic analysis of stone fracture in lithotripsy J Acoust Soc Am 2007 121 2 1190 1202 10.1121/1.2404894 17348540

[B86] da Silva SF R Composition of kidney stone fragments obtained after extracorporeal shock wave lithotripsy Clin Chem Lab Med 2010 48 3 403 404 20113249 10.1515/CCLM.2010.079

[B87] Schuler TD Medical expulsive therapy as an adjunct to improve shockwave lithotripsy outcomes: a systematic review and meta-analysis J Endourol 2009 23 3 387 393 10.1089/end.2008.0216 19245302

[B88] Micali S Can Phyllanthus niruri affect the efficacy of extracorporeal shock wave lithotripsy for renal stones? A randomized, prospective, long term study J Urol 2006 176 3 1020 1022 10.1016/j.juro.2006.04.010 16890682

[B89] Micali S Micali S Efficacy of expulsive therapy using nifedipine or tamsulosin, both associated with ketoprofene, after shock wavw lithotripsy of ureteral stones Urol Res 2007 178 43 89 93 10.1007/s00240-007-0085-5 17396251

[B90] Zheng S Tamsulosin as adjunctive treatmen after shockwave lithitripsy in patients with upper urinary tract stones: a systemic review and meta-analysis Scand J Urol Nephrol 2010 44 425 432 10.3109/00365599.2010.523014 21080841

[B91] Falahatkar S Is there a role for tamsulosin after shock wave lithotripsy in the treatment of renal and ureteral calculi? J Endourology 2011 25 3 495 498 10.1089/end.2010.0439 21166579

[B92] Sighinolfi MC Efficacy of tamsulsin treatment after extracorporeal shock wave lithotripsy of stone located in the kidney: a prospective and randomized study on 129 patient 2010 28° World Congress of ndourology and SWL Abstract

[B93] Kijvikai K Haleblian GE Preminger GM de la Rosette J Shock wave lithotripsy or ureteroscopy for the management of proximal ureteral calculi: an old discussion revisited J Urol 2007 10.1016/j.juro.2007.05.132 17698126

[B94] Aboumarzouk OM Extracorporeal shock wave lithotripsy (ESWL) versus ureteroscopic management for ureteric calculi Cochrane Database Syst Rev 2011 12 CD006029 22161396 10.1002/14651858.CD006029.pub3

[B95] Semins MJ Management of stone disease in pregnancy Curr Opin Urol 2010 20 2 174 177 10.1097/MOU.0b013e3283353a4b 19996751

[B96] Athanasios N Optimizing shock wave lithotripsy in the 21° century Eur Urol 2007 52 344 354 10.1016/j.eururo.2007.04.066 17499914

[B97] Geavlete P flexible ureteroscopy: reshaping the upper urinary tract endourology Arch Esp Urol 2011 64 1 3 13 21289380

[B98] Traxer O Dubosg F Jamali K Gattegno B Thibault P New-generation flexible ureterorenoscopes are more durable than previous ones Urology 2006 68 2 276 279 10.1016/j.urology.2006.02.043 16904434

[B99] Monga M Best S Venkatesh R Ames C Lee C Kuskowski M Shwartz S Vanlangendock R Skepazy J Landman J Durability of flexible ureteroscopes: a randomized, prospective study J Urol 2006 176 1 137 141 10.1016/S0022-5347(06)00575-1 16753388

[B100] Canes D Desai MM New technology in the treatment of nephrolithiasis Curr Opin Urol 18 235 240 18303551 10.1097/MOU.0b013e3282f51949

[B101] Paffen ML A comparison of the physical properties of four new generation flexible ureteroscopes: (de)flection, flow properties, torsion stiffness, and optical characteristics Endourol 2008 22 10 2227 2234 10.1089/end.2008.0371 18831670

[B102] Haberman K A dual-channel flexible ureteroscope: evaluation of deflection, flow, illumination, and optics J Endourol 2011 25 9 1411 1414 Epub 2011 Jul 28 10.1089/end.2010.0642 21797758

[B103] Wendt-Nordahl G Do new generation flexible ureterorenoscopes offer a higher treatment success than their predecessors? Urol Res 2011 39 3 185 188 10.1007/s00240-010-0331-0 21052986

[B104] Multescu R Conventional fiberoptic flexible ureteroscope versus fourth generation digital flexible ureteroscope: a critical comparison J Endourol 2010 24 1 17 21 10.1089/end.2009.0390 19954350

[B105] Jiang H Wu Z Ding Q Zhang Y Ureteroscopic treatment of ureteral calculi with holmium: YAG laser lithotripsy J Endourol 2007 21 2 151 154 10.1089/end.2006.0209 17338611

[B106] Binbay M Evaluation of pneumatic versus holmium: YAG laser lithotripsy for impacted ureteral stones Int Urol Nephrol 2011 43 4 989 995 10.1007/s11255-011-9951-8 21479563

[B107] Hyams ES Flexible ureterorenoscopy and holmium laser lithotripsy for the management of renal stone burdens that measure 2 to 3 cm: a multi-institutional experience J Endourol 2010 24 10 1583 1588 10.1089/end.2009.0629 20629566

[B108] Blew BD Dagnone AJ Fazio LM Practical comparison of four nitinol stone baskets J Endourol 2007 21 655 658 10.1089/end.2007.9959 17638565

[B109] Korman E Comparison of small diameter stone baskets in an in vitro caliceal and ureteral model J Endourol 2011 25 1 123 127 10.1089/end.2010.0312 20977371

[B110] Morris DS Wei JT Taub DA Temporal trends in the use of percutaneous nephrolithotomy J Urol 2006 175 1731 1736 10.1016/S0022-5347(05)00994-8 16600744

[B111] Galvin DJ Pearle MS The contemporary management of renal and ureteric calculi BJU Int 2006 98 6 1283 1288 10.1111/j.1464-410X.2006.06514.x 17125486

[B112] Méndez Probst CE Preoperative indications for percutaneous nephrolithotripsy in 2009 J Endourol 2009 23 10 1557 1561 10.1089/end.2009.1518 19630500

[B113] Valdivia JG Supine versus prone position during percutaneous nephrolithotomy: a report from the clinical research office of the endourological society percutaneous nephrolithotomy global study J Endourol 2011 25 10 1619 1625 10.1089/end.2011.0110 21877911

[B114] Duty B The debate over percutaneous nephrolithotomy positioning: a comprehensive review J Urol 2011 186 1 20 25 Epub 2011 May 14 10.1016/j.juro.2011.02.2693 21571342

[B115] Ibarluzea G Supine Valdivia and modified lithotomy position for simultaneous anterograde and retrograde endourological access BJU Int 2007 100 1 233 236 10.1111/j.1464-410X.2007.06960.x 17552975

[B116] Cracco CM ECIRS (endoscopic combined intrarenal surgery) in the galdakao-modified supine valdivia position: a new life for percutaneous surgery? World J Urol 2011 29 6 821 827 10.1007/s00345-011-0790-0 22057344

[B117] Kawahara TB Ureteroscopy assisted retrograde nephrostomy: a new technique for percutaneous nephrolithotomy (PCNL) JU Int 2011 111 67 123 127 10.1111/j.1464-410X.2011.10795.x 22142188

[B118] Wong C Leveillee RJ Single upper-pole percutaneous access for treatment of > orÂ¼5-cm complex branched staghorn calculi: is shockwave lithotripsy necessary? J Endourol 2002 16 477 481 10.1089/089277902760367430 12396440

[B119] Undre S Olsen S Mustafa N Patel A Pass the ball! Simultaneous flexible nephroscopy and retrograde intrarenal surgery for large residual upper-pole stag horn stone J Endourol 2004 18 844 847 10.1089/end.2004.18.844 15659916

[B120] Ganpule AP Multiperc versus single perc with flexible instrumentation for staghorn calculi J Endourol 2009 23 10 1675 1678 10.1089/end.2009.1535 19715481

[B121] Akman T Comparison of outcomes after percutaneous nephrolithotomy of staghorn calculi in those with single and multiple accesses J Endourol 2010 24 6 955 960 10.1089/end.2009.0456 20443700

[B122] Li HL Percutaneous nephrolithotomy through the upper pole calix access for complicated renal calculi: report of 581 cases Nan Fang Yi Ke Da Xue Xue Bao 2011 31 12 2079 2081 22200717

[B123] Gokten OE Efficacy of levobupivacaine infiltration to nephrosthomy tract in combination with intravenous paracetamol on postoperative analgesia in percutaneous nephrolithotomy patients J Endourol 2011 25 1 35 39 10.1089/end.2010.0346 21067273

[B124] Chen Y Minimally invasive percutaneous nephrolithotomy under peritubal local infiltration anesthesia World J Urol 2011 29 6 773 777 Epub 2011 Jul 22 10.1007/s00345-011-0730-z 21779834

[B125] Bove P Is telesurgery a new reality? Our experience with laparoscopic and percutaneous procedure J Endourol 2003 17 3 137 142 10.1089/089277903321618699 12803985

[B126] Yates D From Leonardo to da Vinci: the history of robot-assisted surgery in urology RBJU Int 2011 108 11 1708 1713 10.1111/j.1464-410X.2011.10576.x 21951677

[B127] Desai MM Aron M Gill IS Pascal-Haber G Ukimura O Kaouk JH Stahler G Barbagli F Carlson C Moll F Flexible robotic retrograde renoscopy: description of novel robotic device and preliminary laboratory experience Urology 2008 72 1 42 46 10.1016/j.urology.2008.01.076 18372029

[B128] Desai MM Robotic flexible ureteroscopy for renal calculi: initial clinical experience J Urol 2011 186 2 563 568 10.1016/j.juro.2011.03.128 21683380

[B129] Mozer P Troccaz J Stoianovici D Urologic robots and future directions Curr Opin Urol 2009 19 1 114 119 10.1097/MOU.0b013e32831cc1ba 19057227 PMC2672590

[B130] Mariani A Ureteroscopic monotherapy of large (>2 cm) renal calculi J Endourol 2008 22 Suppl 1 202

